# Supplementary material for: Comparative multiomics analysis of cell physiological state after culture in a basket bioreactor
Source: Sci Rep. 2022 Nov 23;12:20161. doi: 10.1038/s41598-022-24687-4 (PMC9686226; doi:10.1038/s41598-022-24687-4)

3666068.1:10964158:10964306:+@NW_023666068.1:10964158:10964662:+@NW_023666068.1:10967066:1096

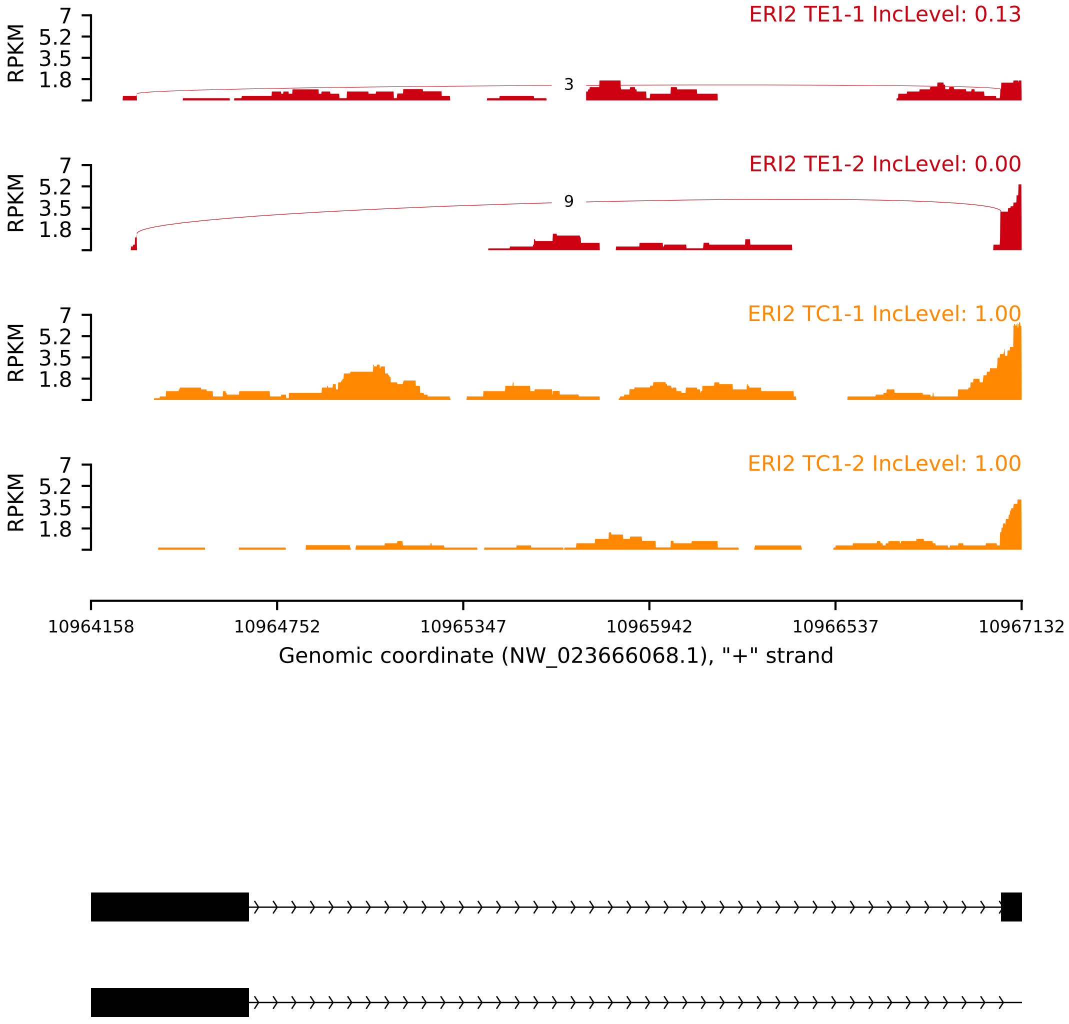

Supplement: Supplementary file 1 — Supplementary Information 1. [file 41598_2022_24687_MOESM1_ESM.zip › raw data/Transcriptomic raw data/7.AS/2.dasplot/TE1vsTC1/A5SS/3_ERI2_NW_023666068.1_10964158_10964306_+@NW_023666068.1_10964158_10964662_+@NW_023666068.1_10967066_10967133_+.pdf]
